# The Use of Fluids in Sepsis

**DOI:** 10.7759/cureus.528

**Published:** 2016-03-10

**Authors:** Audrey A Avila, Eliezer C Kinberg, Nomi K Sherwin, Robinson D Taylor

**Affiliations:** 1 University of Central Florida College of Medicine

**Keywords:** sepsis, fluids, resuscitation, intensive care, colloids, crystalloids

## Abstract

Sepsis is a systemic inflammatory response to severe infection causing significant morbidity and mortality that costs the health care system $20.3 billion annually within the United States. It is well established that fluid resuscitation is a central component of sepsis management; however, to date there is no consensus as to the ideal composition of fluid used for resuscitation. In this review, we discuss the progression of clinical research comparing various fluids, as well as the historical background behind fluid selection for volume resuscitation. We conclude that the use of balanced fluids, such as Ringer’s Lactate, seems very promising but further research is needed to confirm their role.

## Introduction and background

Sepsis is one of the most common conditions in patients admitted to the intensive care unit (ICU) and consists of a systemic inflammatory response to a presumed or proven infection. Between 6–30% of all ICU patients are affected by sepsis, which causes significant morbidity and mortality [1]. In 2011 alone, $20.3 billion dollars was spent on sepsis within the United States [2]. Approximately one million cases of sepsis progress to severe sepsis annually in the United States [3]. Severe sepsis is the most common cause of death in non-coronary ICUs, with approximately one third of patients dying during the course of their hospitalization [4]. If severe sepsis further progresses to septic shock, in-hospital mortality increases from 20–50% to 40–80% [1]. Sepsis causes massive vasodilation and increases membrane permeability leading to an intravascular fluid deficit. As such, much research has been done surrounding intravenous fluid therapy in the setting of sepsis.

A literature review was conducted to determine the ideal intravenous fluid for resuscitation in sepsis. A PubMed search was conducted using the terms “sepsis” and “fluid choice,” with the modifier of articles published within the past ten years; 101 articles met these criteria. Study abstracts were screened for relevance to the clinical topic. Subsequently, full text articles were reviewed to determine their applicability. Articles that compared the use of specific fluid types relative to others, whether in the intensive care unit, hospital, or emergency room setting were selected.

A landmark study in 2001 by Rivers et al., demonstrated improved mortality and decreased length of hospital stay in severe sepsis and septic shock due to a concept they entitled early goal-directed therapy (EGDT) [5]. EGDT utilizes continuous monitoring of central venous pressure, mean arterial pressure, and central venous oxygen saturation to guide use of intravenous fluids, vasoactive drugs, and red-cell transfusions in order to optimize tissue oxygen delivery. In 2002, the Surviving Sepsis Campaign was formed to develop uniform, current, and evidence-based guidelines for management of severe sepsis and septic shock. The first guidelines were published in 2004 and highlighted the need for aggressive fluid resuscitation, as have all revisions of the guidelines since then [2]. However, in 2004, it was felt that fluid resuscitation could be with either colloids or crystalloids due to a lack of large-scale meta-analyses and randomized controlled trials comparing resuscitation with crystalloids and colloids. This resulted in a surge of research into the ideal fluid for resuscitation in sepsis, which is reflected by the most recent guidelines published in 2012. The update recommends crystalloids as the initial choice for fluid resuscitation in sepsis with albumin as an adjuvant when patients require substantial amounts of crystalloids [2]. However, there is still no recommendation as to the type of crystalloid to use.

Broadly, fluids can be divided based on their composition into crystalloids and colloids. Colloids are substances that do not diffuse through membranes as easily as crystalloids, and thus are confined to the intravascular space. When administered to a patient with sepsis, colloids exert an osmotic effect leading to the retention of fluid in the intravascular compartment. Table 1 [6-7] compares the composition and characteristics of various colloids. Crystalloid solutions are composed of molecules that can diffuse across cell membranes, and can be divided into balanced and non-balanced formulas. Non-balanced fluids, such as normal saline (NS), are isotonic to plasma but do not contain the equivalent electrolyte composition. Balanced crystalloids, such as Ringer's Lactate, Ringer’s Acetate, and PlasmaLyte are developed to resemble the composition of plasma. Table 2 [6-8] compares the composition of these fluids to plasma.

**Table 1:** Composition of Colloids

	Osmolality (mOsm/L)	Oncotic Pressure (mmHg)	Plasma Expansion (%)	Duration of Plasma Expansion (hr)	Plasma Half-life (hr)	Electrolytes (mEq/L)		Price ($/ L)
						Na+	Cl-	
HES; 6% Pentastarch; 10% Pentastarch	300-326	23-82	100-160	<12	2-12	154	154	31.30
4% Albumin	300	19-30	70-100	<24	16-24	140	128	92.84

**Table 2:** Composition of Crystalloids

	Osmolality (mOsm/L)	pH (mol/L)	Electrolytes (mEq/L)					Buffers (mEq/L)	Price ($/L)
			Na^+^	Cl^-^	K^+^	Ca^2+^	Mg^2+^		
Plasma	290	7.4	140	103	4	4	2	Bicarbonate (24)	N/A
0.9% saline	308	5.7	154	154	0	0	0	N/A	1.03
Hartmann’s solution	279	6.5	131	111	4	2	0	Lactate (29)	0.94
Ringer’s Lactate	273	6.5	130	109	4	3	0	Lactate (28)	0.94
Ringer’s Acetate	275	6.7	131	109	4	3	0	Acetate (28)	*
PlasmaLyte	295	7.4	140	98	5	0	3	Acetate (28) Gluconate (23)	2.21

The administration of various IV fluids began during the cholera epidemic in 1831, with saline-based solutions being first administered by Thomas Latta, who described the injections as “restoring the natural current in the veins and arteries, of improving the colour of the blood, and recovering the functions of the lungs” [9]. Latta’s original solution would be considered a balanced crystalloid, as it contained 134 mmol/L Na+, 188 mmol/L Cl- and 16 mmol/L of HCO3^-^. In the mid-19^th^ century, significant research focused on the composition of salt solutions, which lead to the development of Ringer’s solution, the precursor to modern-day Ringer’s Lactate. In the late 19^th^ century, there was an understanding that an ideal solution needed to be isotonic with serum and in 1896, Hamburger suggested a concentration of 0.92% saline as normal for the blood of mammals based on comparison of the “freezing point” of serum between humans and animals and *in vitro* studies of red blood cells [9]. His research prompted the development of 0.9% saline, which is one of the most commonly used IV fluid today [9].

Fluid composition can have unintended physiological effects, such as altering the pH balance. The metabolism of organic anions such as lactate and acetate leads to the production of bicarbonate. Therefore, these solutions can cause metabolic alkalosis if the metabolizable anions are infused in concentrations that exceed the lack of bicarbonate. Problems associated with the use of Ringer’s Lactate include the inability to use lactate as a marker of hypoxia and the fact that lactate may increase oxygen consumption [10]. Furthermore, it cannot be used in patients with lactic acidosis and is often contraindicated in septic patients, who have a hepatic disruption of lactate clearance. Ringer’s Acetate is an alternative balanced crystalloid that may be used in patients with diabetic lactic acidosis and those with impaired hepatic metabolism [10]. Normal saline is a non-balanced crystalloid that has a lower ratio of Na:Cl than balanced crystalloids and can result in hyperchloremic non-anion gap metabolic acidosis [11]. These factors, along with many others, must be considered when selecting a fluid for volume resuscitation.

Due to the serious and prevalent nature of sepsis, and the multitude of available fluid options, extensive research has been conducted to identify optimal treatment protocols. The Campaign’s guidelines have been revised three times since its initial publication, and have incorporated many aspects of EGDT to reflect the current literature. Ultimately, fluid selection in today’s intensive care units for treatment of sepsis is often influenced by physician preference, hospital protocol, and regional availability due to a lack of evidence-based guidelines on specific fluid choice.

## Review

### Crystalloids versus colloids       

Our review focuses on differentiating fluids by discussing individual colloid options, comparing crystalloids versus colloids, and finally evaluating current research concerning balanced and non-balanced crystalloids.

Colloids themselves form a broad group, and include albumin, hydroxyethyl starch (HES), dextrans, and gelatins. Many studies, including the Efficacy of Volume Substitution and Insulin Therapy in Severe Sepsis (VISEP) trial, Scandinavian Starch for Severe Sepsis/Septic Shock (6S) trial, and Crystalloid vs. Hydroxy-Ethyl Starch Trials (CHEST) were conducted to compare the efficacy of each in septic patients. It was found that there was no significant difference in the effectiveness and adverse effects of different colloids. In fact, only albumin has been found to be neutral or beneficial [12], and some colloids were proven to be dangerous [13]. For example, HES is associated with acute renal failure [14], need for renal replacement therapy [14-15], bleeding, and even increased mortality [16]. Though data about gelatin is scant, one study found it to be associated with increased mortality [17]. As such, the only colloid that should be considered for clinical use is albumin.

A 2011 article by Delaney et al. assessed the use of albumin solutions at different concentrations and utilized death as a primary outcome [12]. The article analyzed eight studies covering 383 patients who were treated with concentrated solutions of >20% albumin, and nine studies covering 1594 patients that used more dilute solutions (4%, 4.5%, and 5% albumin). The pooled estimate of the odds ratio for death when compared to other fluid treatments was 1.08 in the concentrated albumin group, as compared to 0.76 in the dilute albumin group [12]. This data seems to indicate that increased colloid concentration is associated with improved clinical outcomes. Postulated theories for the apparent beneficial effects of albumin in sepsis include the intravascular volume expansion it affords, its ability to transport biologically active molecules, maintain colloid osmotic pressure, maintain the permeability of the capillary membrane, and act as a free radical scavenging antioxidant.

Taken as a whole, these three trials failed to demonstrate a clear benefit in the use of colloids in sepsis rather than crystalloids. Given the cost of albumin and safety issues related to some other colloids, the sepsis resuscitation fluid debate has moved toward identifying the optimal crystalloid choice for use in sepsis. This shift may further benefit patients, as crystalloids are more readily available and are cheaper than colloid solutions.

In 2004, the Saline versus Albumin Fluid Evaluation (SAFE) study was the first large-scale randomized controlled trial (RCT) to examine the association between fluid choice and survival in ICU patients [4]. This trial compared NS to 4% albumin as monotherapy for fluid resuscitation and maintenance. Patients were randomized to one fluid group, and were treated with that fluid until death, discharge, or 28 days after initiation of the trial. The primary outcome was death from any cause at 28 days, sub-grouped by trauma, severe sepsis, and the presence of acute respiratory distress syndrome (ARDS). They found no significant difference in 28-day mortality across the three groups (20.9% in albumin group and 21.1% of NS group). Furthermore, there was no statistically significant difference between the groups when looking at severe sepsis alone [4].

In 2013 the Colloids Versus Crystalloids for the Resuscitation of the Critically Ill (CRISTAL) study employed a multicenter RCT to compare mortality in ICU patients resuscitated with crystalloids (saline or Ringer’s Lactate) versus colloids (gelatins, dextrans, hydroxyethyl starches, or albumin) [18]. Although there was no statistically significant difference in mortality 28 days after initiation of therapy, the study tracked patients until 90 days after onset of therapy and found significantly fewer deaths in the colloids group as compared to the crystalloids group (30.7% vs 34.2%, RR 0.92 [95% CI, 0.86–0.99], P = .03). The colloid group also experienced fewer days of mechanical ventilation, and fewer days of vasopressor therapy. However, it is important to note this trial contained a significant flaw: many patients were treated prior to enrollment in this study and 30–50% received a fluid different than the one to which they were ultimately randomized during the time prior to enrollment. Furthermore, both groups of patients received isotonic crystalloid for maintenance fluid. Thus, while the results were significant, caution should be used when interpreting the results due to the flaws inherent in the methodology [18].

The Albumin Italian Outcome Sepsis  (ALBIOS) trial in 2014 was a large RCT which compared administration of crystalloid to administration of 20% albumin with crystalloid [5]. No distinction was made between the type of crystalloid, be it balanced or unbalanced, within both groups. The primary outcome was death at 28 days [5]. At 28 days, 31.8% of patients in the albumin group and 32.0% of patients in the crystalloid group had died (RR 1.00; 95% CI, 0.87–1.14; P=.94). Similarly, at 90 days, 41.1% of the albumin group and 43.6% of the crystalloid group had died (RR 0.94; 95% CI, 0.85–1.05; P= 0.29). Thus, there was no significant difference between the two groups at either 28 or 90 days after initiation of therapy [5].

### Choice of crystalloid         

A 2014 article from Zhou et al. investigated the effect of NS and PlasmaLyte on acute kidney injury (AKI) in rats with sepsis [19]. When using the Risk, Injury, Failure, Loss of kidney function, and End-stage kidney disease (RIFLE) criteria to assess the extent of kidney injury, Zhou found that treatment with NS resulted in significantly worse kidney function as compared to balanced crystalloids (83% vs 28%, p <.001) [19]. Furthermore, pathological analysis revealed more conspicuous damage to renal tubules in rats resuscitated with NS compared to PlasmaLyte. Despite these findings in the rat population, a recent human trial conducted in four New Zealand ICUs failed to show any difference in the rates of AKI in patients who received NS versus balanced crystalloids [20]. Of note, this study did not have strict inclusion or exclusion criteria, and many patients received NS before being transferred to the ICU and being enrolled in the study.

A systematic review and meta-analysis done by Rochwerg et al. in 2014 looked specifically at types of fluid used for resuscitation in sepsis [17]. A significant amount of the research has been restricted by including a broader patient population such as intensive care unit patients in general or by focusing on only a few types of fluid, so this meta-analysis used and re-analyzed the available date. In total, this included 14 randomized controlled trials with a total of 18,916 patients and found a slight benefit with regards to mortality of balanced crystalloids when compared to normal saline (0.78, CI = 0.58–1.05) [17]. Furthermore, they found that balanced crystalloids had similar mortality outcomes to albumin (0.95, CI = 0.65–1.38) [17]​. Though the authors admit that they did not have many studies on which to base their results, their results are important as, unlike most other studies and reviews, this review focuses solely on fluid resuscitation specifically in sepsis [17].

In December 2015, Raghunathan et al. published the first study comparing balanced and non-balanced crystalloids in a human population being treated for sepsis. They utilized a retrospective cohort study of 60,734 ICU patients with severe sepsis or septic shock and assessed the use of NS, NS with balanced crystalloids, NS with colloids, and a combination of all three together [21]. Their population was limited to those who held a primary or secondary diagnosis of sepsis, and received at least two liters of fluids and vasopressors by the hospital day two. Surgical patients were excluded. The primary outcome focused on in-hospital mortality, while secondary outcomes were length of stay and cost. Administering a combination of NS and balanced crystalloids together resulted in a mortality of 17.69%, which was significantly lower than the mortality rate of patients treated with NS as monotherapy (20.19%) or NS with colloids (24.16%). A combination of NS, balanced crystalloid, and colloids resulted in a similar mortality rate to NS and balanced crystalloids without the addition of colloids (19.23%). The study did not reveal a significant difference in total cost or length of stay in the NS monotherapy group versus NS and balanced crystalloids. One of the key findings identified by the authors was that “administration of balanced crystalloids by hospital day two was consistently associated with lower mortality, whether colloids were used or not” [21].

Studies conducted in additional populations have also demonstrated a benefit of balanced crystalloid as compared to NS. A 2012 study by Shaw et al. compared the use of NS vs PlasmaLyte during open abdominal surgery, and focused on major post-surgical morbidity [22]. Primary outcomes included respiratory failure persisting for more than 24 hours post-operation, cardiac complications requiring intervention, gastro-intestinal bleeding, infection, and acute renal failure. Secondary outcomes included electrolyte disturbances, acidosis, and re-hospitalization. The study included 30,994 patients in the NS group and 926 in the PlasmaLyte group. Patients who received PlasmaLyte had significantly lower rates of post-op infections (OR = 0.681, CI = 0.505–0.981), renal failure leading to dialysis (1.0% vs 8.3%, p <.001), blood transfusions (1.8% vs 13.3%, p <.001), and electrolyte instabilities (OR = 0.755, CI = 0.595–0.958) [22].

Young et al. published a randomized controlled trial in 2014 comparing the use of PlasmaLyte to NS for initial resuscitation of 46 trauma patients [23]. The primary outcome was base excess, an established marker for adequate resuscitation in the setting of trauma, in the first 24 hours, with secondary outcome measures including serum electrolyte levels, osmolality, and arterial pH^23^. Fluids were titrated to meet parameters for blood pressure, CVP, resolution of acidosis, and urine output. Analysis revealed that PlasmaLyte was associated with a quicker and more persistent clearance of base deficit as compared to NS. Further, urine output in the balanced crystalloid group was significantly higher than the NS group, indicating better end-organ perfusion and reduced kidney injury  [23].

## Conclusions

The subject of which fluid should be used in sepsis has remained a hotly contested topic. Due to the results of the SAFE, CRISTAL, and ALBIOS trials that did not show a clear benefit associated with the use of albumin, increased cost of albumin, and safety issues with use of HES, crystalloids appear to be the fluid of choice for patients with a diagnosis of sepsis. As there has been no evidence-base for choice of crystalloid in the past, it is likely that people are comfortable using NS based on routine clinical practice. Recent research shows promising results with the use of balanced crystalloids, although further large-scale RCTs comparing balanced and non-balanced solutions are needed. It is possible that physicians may not be as familiar with the use of balanced solutions and the various options that are available. Further, there may be a misconception that NS is more affordable than balanced solutions, but Ringer’s Lactate and Hartman’s solution are actually cheaper than NS, as shown previously in Table 2. Due to the importance of fluids in the resuscitation of sepsis patient, it is imperative that physician decision making in this regard be guided by a strong evidence base. We contend that there is a need for further research on the use of crystalloids to develop evidence-based guidelines for future clinical decision making.
